# A study of spatial resolution in pollution exposure modelling

**DOI:** 10.1186/1476-072X-6-19

**Published:** 2007-06-04

**Authors:** Emilie Stroh, Lars Harrie, Susanna Gustafsson

**Affiliations:** 1 GIS Centre, Lund University, Sölvegatan 12, SE-223 62 Lund, Sweden; 2 Malmö Evironmental Protection Agency, Malmö Municipality, Bergsgatan 17, SE-205 80 Malmö, Sweden

## Abstract

**Background:**

This study is part of several ongoing projects concerning epidemiological research into the effects on health of exposure to air pollutants in the region of Scania, southern Sweden. The aim is to investigate the optimal spatial resolution, with respect to temporal resolution, for a pollutant database of NO_x_-values which will be used mainly for epidemiological studies with durations of days, weeks or longer periods. The fact that a pollutant database has a fixed spatial resolution makes the choice critical for the future use of the database.

**Results:**

The results from the study showed that the accuracy between the modelled concentrations of the reference grid with high spatial resolution (100 m), denoted *the fine grid*, and the coarser grids (200, 400, 800 and 1600 meters) improved with increasing spatial resolution. When the pollutant values were aggregated in time (from hours to days and weeks) the disagreement between the fine grid and the coarser grids were significantly reduced. The results also illustrate a considerable difference in optimal spatial resolution depending on the characteristic of the study area (rural or urban areas). To estimate the accuracy of the modelled values comparison were made with measured NO_x _values. The mean difference between the modelled and the measured value were 0.6 μg/m^3 ^and the standard deviation 5.9 μg/m^3 ^for the daily difference.

**Conclusion:**

The choice of spatial resolution should not considerably deteriorate the accuracy of the modelled NO_x _values. Considering the comparison between modelled and measured values we estimate that an error due to coarse resolution greater than 1 μg/m^3 ^is inadvisable if a time resolution of one day is used. Based on the study of different spatial resolutions we conclude that for urban areas a spatial resolution of 200–400 m is suitable; and for rural areas the spatial resolution could be coarser (about 1600 m). This implies that we should develop a pollutant database that allows different spatial resolution for urban and rural areas.

## Background

The air we breathe contains a vast number of chemical pollutants. Some originate from natural sources such as soil or salt particles generated from nearby fields or the sea, but most of them originate from man-made sources such as vehicles, industrial plants, combustion and heating. These chemicals not only interact with each other but also with the cells and compounds within our respiratory and cardiovascular systems. Studies have shown that exposure to air pollutants such as fine particles and nitrogen dioxide contributes to excess diseases and mortality among people suffering from cancer, respiratory and cardiovascular diseases [1]. It has also been shown that air pollution leads to poorer health among vulnerable groups such as asthmatics, old people and children. The health effects on children are of special concern since these health problems might have repercussions later in life [2].

Geographic analyses have previously been used in epidemiology to study the correlation between exposure to air pollutants and health [3,4]. Some of these analyses were conducted in three steps: (1) modelling of the pollutant field using a dispersion model and an emission source database, (2) using the pollutant field to estimate the exposure to pollutants of the study group, and (3) correlation of the exposure with health status of the study group.

This study is part of several ongoing projects involving epidemiological research in the region of Scania in southern Sweden. These projects employ population databases obtained from the Regional Office of Scania and are based on the Swedish National Population Register. These population databases are comparatively detailed regarding both the high degree of detail in the socio-economic and health data they contain, but also in their spatial resolution. All the databases contain information on individuals, including the exact location of their residence. This location is either represented as a point at the centre of the real estate where they live, or as a centroid in a 500 m-1 km grid. This detailed information regarding the population in Scania enables us to perform exposure studies with high resolution.

An emission source database including traffic, industry, and combustion was established and dispersion modelling software implemented [5]. However, it was found to be impractical to conduct dispersion modelling for each epidemiological study. Therefore, we plan to establish a *pollutant database of *NO_x _values; this database will contain pollutant values in a fixed grid covering Scania for the time period 2000–2004. The advantage of a *pollutant database*, rather than an *emission source database*, is that it will make epidemiological studies much easier to perform for users who are not accustomed to using emission source databases and dispersion modelling. The disadvantage is that a pollutant database is not as flexible as an emission source database, mainly in that the pollutant database has a fixed resolution in time and space.

It is important to take the time factor into consideration when conducting geographical analysis in exposure studies with the aim of analysing diseases and health hazards with long latency periods, such as cancers. Samples and measurements made when a disease manifests itself do not usually represent historical exposure levels [6]. It is therefore important to collect data in order to be able to model accumulated exposure levels from previous years. This need to store data raises the issues of storing capacity and modelling resources. Since digital data for these studies are often required as high-resolution data with a low level of spatial aggregation, the storage capacity needed and the modelling time required increase rapidly. This problem is apparent in exposure studies that take the movement of a dynamic population into account; not only do these studies demand digital data with high spatial resolution, but there is also a need for high temporal resolution.

The aim of this study was to examine the optimal spatial resolution, with respect to the temporal resolution, for our pollutant database. This was done through comparing the absolute differences and standard deviations of the differences among pollution estimates of different spatial and temporal resolutions. The choice of optimal spatial resolution was also related to the accuracy of the estimated pollutant field generated by the dispersion model and the emission database.

### Related studies

The impact of scale and aggregation factors in epidemiological analysis can not be ignored. As Krieger states: "in epidemiological studies *completeness *in geocoding does not equal *success*. Accuracy – and choice of geographic level – matter as much, if not more" [7]. Cromley also concludes that, in many health studies, the aggregated level of available health data is a major limitation since data are often aggregated inappropriately for the purpose of the study [6]. A study by João shows that scale, in terms of both detail and extent, is an important issue, which to date has been largely neglected in environmental impact assessments [8]. This is further emphasized by Setton et al. who stated that many exposure assessments and epidemiological analyses of the impacts of air pollution on health have been undertaken on regional scales, and that only recently have researchers begun to investigate neighbourhood-level variations in pollutant levels [9].

Finding the optimal scale for studies concerned with factors varying in space is a complex matter of great concern. If large areas are defined they reveal more global structures, while local variations are obscured [10]. This means that only superficial assessments may be possible, and the uncertainty will increase [11]. However, although small neighbourhood sizes yield detailed patterns and a more detailed examination may be feasible, the understanding of the broad context may be lost [10,11]. As a result, the choice of optimal size is important when investigating ecological associations with diseases, since these patterns might otherwise be concealed or misjudged. As Gregorio et al. concludes: "spatial analysis results may be rightfully considered conditional upon the particular geography selected for the study" [12].

Apart from the risk of altering the results of a study by choosing the "wrong" scale or aggregation level Ali et al. also concluded that the optimal neighbourhood size is data dependent [10]. Their study suggests that, depending on the factors and correlations included, different neighbourhood sizes may be optimal for different variables. This is supported by findings from our previous study [13]. Therefore, it can not always be assumed that the choice of study area for one set of parameters is optimal for another, even though they may be similar. According to Gregorio et al. nationwide efforts to promote regional health information networks/organisations that cross traditional geo-political boundaries demand a greater understanding of how aggregating health and population data may affect the analysis and interpretation of disease patterns [12].

Due to the four-dimensional nature of the distribution of atmospheric pollutants, the importance of scale and aggregation level applies not only to spatial but also temporal variations. According to Pénard-Morand et al., who conducted a study on schoolchildren's exposure to traffic-related air pollution in France, there is extreme variability in the level of exposure that exists from one place to another [14]. Briggs also concludes that pollution surfaces in urban areas are often extremely complex, with steep gradients away from ground-level sources, and often highly localised hot-spots and peaks [4]. Also, Cyrys et al. state that several studies have documented important variations in pollutant concentrations within cities, especially related to motorised traffic and location within the city – for example city centre versus suburbs [15]. These variations are not only variations in space, but also in time mostly due to human behaviour such as rush-hour traffic and industrial activities. Results from a study by Wentz et al. suggest that there is a distinct relationship between levels of CO_2 _and spatial patterns of human activities in urban areas [16]. The results of their study also show that the temporal and spatial patterns of CO_2 _levels correspond closely to the density of traffic, population and workplaces. Consequently, the optimal choice of resolution for a pollutant database may be mainly dependent on the spatial distribution of the concentration of air pollution in the area, but also on the temporal distribution.

João shows in her study that the chosen scale should be considered as part of the observation bias since the choice of scale, both in the meaning of the details analysed and the spatial extent of study area, can have important repercussions on the results, such as the determination of impact significance and the measurement of environmental parameters [8]. It is therefore of fundamental importance to define the scale of observation. In other words, scale choice should be explained, justified and explicitly stated in all environmental impact assessment studies [8].

### Objectives

The overall objective of this study was to determine the optimal spatial resolution for a pollutant database for the county of Scania, southern Sweden. The database will contain concentration values of nitrogen oxides (NO_x_), and possible also other pollutants, over a period of five years (2000–2004).

The optimal spatial resolution is dependent on which time resolution is considered in the epidemiological study. Since the pollutant database will be used mainly for epidemiological studies with durations of days, weeks or even longer periods, we have evaluated the results using different time resolutions.

The difference between modelled and measured values, i.e. the error estimate, of air pollutant concentrations has also been evaluated since this in combination with the error estimate generated from the choice of resolution will give the total error estimate for the pollutant database.

### Study area

The area studied was Scania, the southernmost county of Sweden. The county covers around 11,350 km^2^, which is approximately 2% of the total Swedish land area. The region is relatively densely populated, with more than 1.1 million people living in the area, which constitutes approximately 11% of the total Swedish population. In Scania, approximately 67% of the population lives close to the west coast. Most road traffic (passenger cars as well as trucks) from the European continent to Sweden and Norway passes through this area, and five motorways run through the region. There are also several harbours in the region and a considerable amount of cargo shipping and ferry transport along the coast. These factors, and the closeness to Copenhagen in Denmark, and the European continent, contribute to high concentrations of air pollutants in the region, compared with most other regions in Sweden.

**Figure 1:**
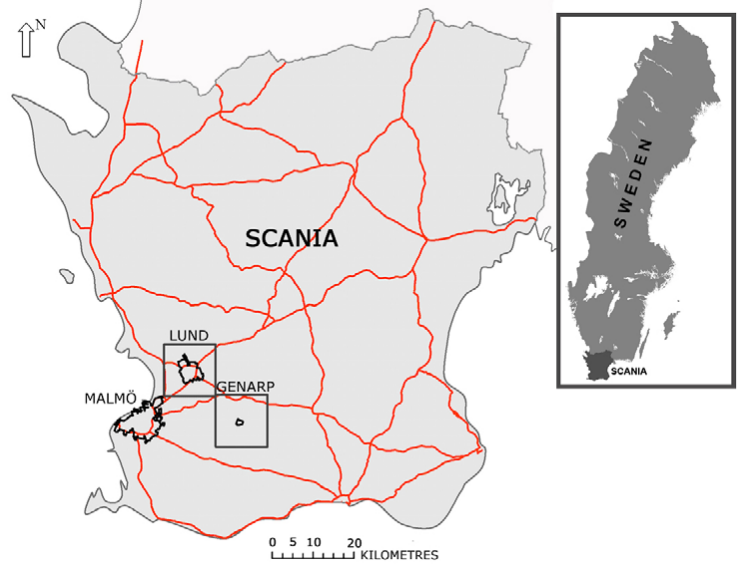
Map showing the two study sites: Lund (urban area) and Genarp (rural area), the location of Malmö, the major roads in the county and Scanias location in Sweden.

We studied two sites in Scania, one urban and one rural site (Figure 1). Both of the study sites were 12.8 × 12.8 km. The urban study site covers the city of Lund and its surroundings, while the rural study site covers the area of a small village called Genarp, located approximately 15 km south-east of Lund.

Lund is the third largest city in Scania with approximately 76,000 inhabitants, and the city covers approximately 23 km^2^. Although the city centre consists mainly of narrow streets and pedestrian precincts, the traffic can be quite intense in some areas, and there are one motorway and two major roads along the north, south and east boundaries of the city. This creates rather steep gradients in the concentrations of traffic-generated pollutants between the city, enclosed by the motorways, and the surroundings, which consist mainly of agricultural land.

The area around Genarp consists mainly of rural and agricultural land with small clusters of buildings. A major road with a large traffic volume passes through the northern part of this study area.

The validation analysis between modelled concentrations and measured concentrations was performed using pollution data obtained from the meteorological station in Malmö (Figure 1). Malmö is the largest city in Scania with a population of about 260,000. The city is located on the west coast facing Denmark and covers an area of 66 km^2^. Malmö is surrounded by a ring road at which three of the five main motorways in the area converge.

### Study design

**Figure 2:**
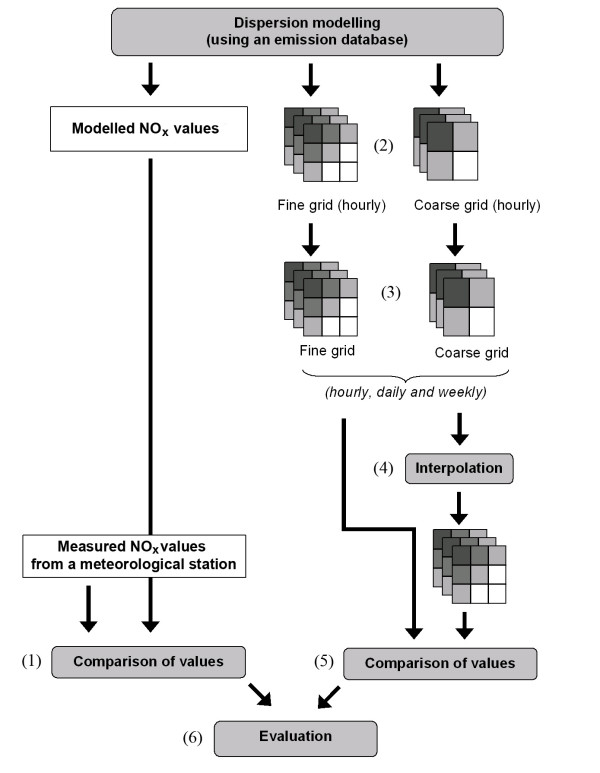
Main flow sheet of the study. The numbers refer to the different steps in the study design.

To investigate suitable spatial resolution for our pollutant database we modelled pollutant fields (NO_X _values) for several spatial and time resolutions. This enabled us to compare the loss of information when using coarser resolutions. This loss of information should be seen in relation to the accuracy of the modelled values. To estimate this quality we compared modelled values with values measured at the meteorological station. Our approach consisted of the following steps (Figure 2).

#### 1) Comparison of modelled values and observed values

Measured hourly NO_X _values from the meteorological station in Malmö city were collected. These measured values were then compared with hourly NO_X _values modelled for the same period and area using a dispersion model and an emission database. Temporal variation, mean and standard deviation was evaluated.

#### 2) Generation of pollutant fields with a time resolution of one hour

A dispersion model was used to estimate pollutant fields. The pollutant field with the highest spatial resolution (100 metre) is below denoted the *fine grid*. The other four grids are denoted *coarse grids *(200, 400, 800 and 1600 metres). Hourly NO_X _values for four weeks were estimated for all grids.

#### 3) Generation of pollutant fields with a time resolution of one day and one week

Mean values of the original hourly data were used to create new pollutant fields with time resolutions of *one day *and *one week *for the time period modelled in step 2.

#### 4) Interpolation of the coarse pollutant fields

The hourly, daily and weekly time series of the coarse grids were interpolated to obtain the same spatial resolution as the fine grid.

#### 5) Comparison of the fine grid and the coarse grids

The values of the fine grid and the interpolated coarse grids were plotted in time series and evaluated concerning the loss of information due to using a coarser spatial resolution in relation to the time resolution (hour, day and week). The spatial distribution of the difference between the grid values was also studied.

#### 6) Evaluation

The results obtained from Step 1 and 5 were evaluated and the spatial resolution of the pollutant database was determined.

## Results and discussion

### Comparison with measured values

**Figure 3:**
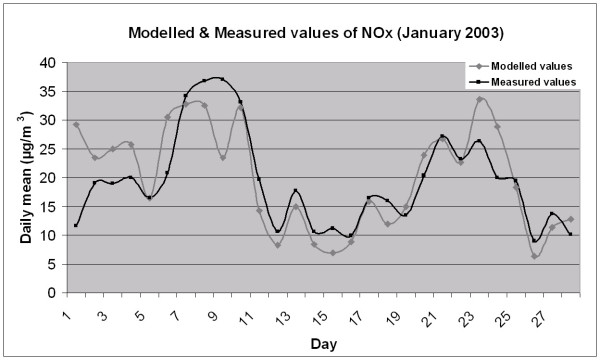
Modelled and measured NO_X _values for the metrological station in Malmö.

To estimate the quality of the modelled concentrations of NO_x_, measured values from the meteorological station in Malmö were compared with the concentration values modelled with the same emission database and dispersion model used to generate values for the study sites. The results were plotted as a time series of daily mean values (Figure 3). As can be seen in the figure, the divergence between the daily time series is large during the first week, and thereafter follows the pattern of the measured values, with some exceptions, reasonably well.

The mean difference between the modelled and the measured value of NO_x _is 0.6 μg/m^3 ^and the standard deviation of the differences is 12.3 μg/m^3 ^for the hourly difference, 5.9 μg/m^3 ^for the daily difference and 4.4 μg/m^3 ^for the weekly difference.

Despite the temporal variations seen in Figure 3 the mean difference is low, with a value of approximately 0.6 μg/m^3 ^NO_x_. However, the standard deviation of the differences reveals that the divergence for the hourly values is high. This number decreases considerably when comparing the concentrations as daily or weekly means. This indicates that the general trend predicted by the dispersion model follows the actual pattern of concentrations of air pollutants in Malmö, but that the dispersion model is too coarse to reflect finer temporal variations than daily means. If the highest and lowest quartiles are removed from the dataset the standard deviation of the means is lowered considerably, and in the case of daily means the standard deviation of the difference is reduced from 5.9 μg/m^3 ^to 2.4 μg/m^3 ^NO_x_.

The main contribution to both the modelled and measured values is traffic generated. The dispersion model uses predefined patterns of traffic flow in the area to estimate the concentration and levels of emitted air pollutants. This may be the reason for the large divergence between modelled and measured values at the beginning of the month (days 1–6). During the first week in January many people are still on holiday due to the Christmas and New Year holidays, causing the traffic flow to diverge from its normal pattern. This will probably lead to lower levels of measured traffic-generated emissions than the modelled results. However, this does not explain the large divergence between modelled and measured values during days 9–11 and 23–26, which probably originates, from errors related to emission sources or the calculation of the dispersion model.

Due to limited possibilities to measure actual levels of NO_X _at the two study sites (Lund and Genarp) the comparison between modelled and measured NO_X _levels for the two study areas should be considered with care. The difference in spatial characteristics, such as emission sources, between Malmö and the two study sites might cause the evaluation of the accuracy and performance of the dispersion model to be invalid for Lund and Genarp. Nevertheless this meteorological station was the closest one to our study areas and the accuracy of the dispersion model and emission database used have been evaluated for the whole county of Scania elsewhere [5] with good agreement. Subsequently we consider this comparison to be a good indication of the general agreement between the validity of the emission database and the performance of the dispersion modelled compared to actual levels of NO_X_.

### Comparison of interpolation methods

**Figure 4:**
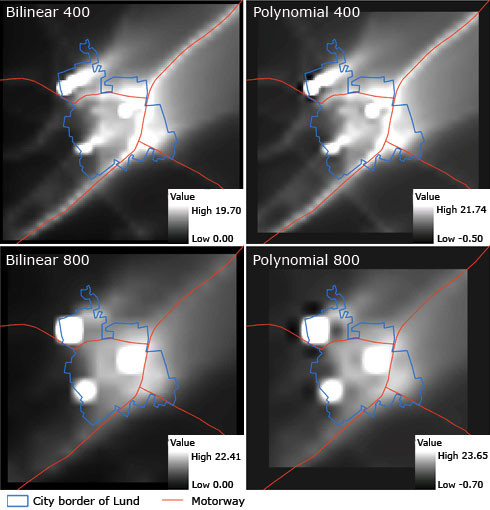
NO_X _values [μg/m^3^] obtained by bilinear and polynomial interpolation for the urban study site with spatial resolution 400 and 800 metres and a time resolution of one hour during the whole of January 2003. The polynomial interpolation method requires 16 (4 × 4) input values, unlike the bilinear interpolation method, which requires only 4 input values, and therefore a large proportion of the grid cells close to the boundary of the modelled area are not computed.

Coarse grids (with spatial resolutions of 200, 400,800 and 1600 m) of hourly data were interpolated by bilinear interpolation (Eq. 1) and polynomial interpolation (Eq. 2) for the urban study site. The mean and standard deviation of the difference between interpolated grids and the fine grid (100 m resolution) were computed. In Table 1 the mean values for all cells given by Eq. 5 are listed. Figure 4 illustrates the interpolated daily mean of NO_x_, during January, calculated for the urban study site (originating from the 400 and 800 resolutions).

**Table 1:** Mean (Δ¯¯
MathType@MTEF@5@5@+=feaafiart1ev1aaatCvAUfKttLearuWrP9MDH5MBPbIqV92AaeXatLxBI9gBaebbnrfifHhDYfgasaacH8akY=wiFfYdH8Gipec8Eeeu0xXdbba9frFj0=OqFfea0dXdd9vqai=hGuQ8kuc9pgc9s8qqaq=dirpe0xb9q8qiLsFr0=vr0=vr0dc8meaabaqaciaacaGaaeqabaqabeGadaaakeaadaqdbaqaaiabfs5aebaaaaa@2E24@) and standard deviation (s¯hour
 MathType@MTEF@5@5@+=feaafiart1ev1aaatCvAUfKttLearuWrP9MDH5MBPbIqV92AaeXatLxBI9gBaebbnrfifHhDYfgasaacH8akY=wiFfYdH8Gipec8Eeeu0xXdbba9frFj0=OqFfea0dXdd9vqai=hGuQ8kuc9pgc9s8qqaq=dirpe0xb9q8qiLsFr0=vr0=vr0dc8meaabaqaciaacaGaaeqabaqabeGadaaakeaacuWGZbWCgaqeamaaBaaaleaacqWGObaAcqWGVbWBcqWG1bqDcqWGYbGCaeqaaaaa@33FF@) of the difference between interpolated and modelled hourly NOX values for the whole urban study site, January 2003.

**Resolution (m)**	**Bilinear **Δ¯¯ MathType@MTEF@5@5@+=feaafiart1ev1aaatCvAUfKttLearuWrP9MDH5MBPbIqV92AaeXatLxBI9gBaebbnrfifHhDYfgasaacH8akY=wiFfYdH8Gipec8Eeeu0xXdbba9frFj0=OqFfea0dXdd9vqai=hGuQ8kuc9pgc9s8qqaq=dirpe0xb9q8qiLsFr0=vr0=vr0dc8meaabaqaciaacaGaaeqabaqabeGadaaakeaadaqdbaqaaiabfs5aebaaaaa@2E24@**[ug/m^**3**^]**	**Bilinear **s¯hour MathType@MTEF@5@5@+=feaafiart1ev1aaatCvAUfKttLearuWrP9MDH5MBPbIqV92AaeXatLxBI9gBaebbnrfifHhDYfgasaacH8akY=wiFfYdH8Gipec8Eeeu0xXdbba9frFj0=OqFfea0dXdd9vqai=hGuQ8kuc9pgc9s8qqaq=dirpe0xb9q8qiLsFr0=vr0=vr0dc8meaabaqaciaacaGaaeqabaqabeGadaaakeaacuWGZbWCgaqeamaaBaaaleaacqWGObaAcqWGVbWBcqWG1bqDcqWGYbGCaeqaaaaa@33FF@**[ug/m^**3**^]**	**Polynomial **Δ¯¯ MathType@MTEF@5@5@+=feaafiart1ev1aaatCvAUfKttLearuWrP9MDH5MBPbIqV92AaeXatLxBI9gBaebbnrfifHhDYfgasaacH8akY=wiFfYdH8Gipec8Eeeu0xXdbba9frFj0=OqFfea0dXdd9vqai=hGuQ8kuc9pgc9s8qqaq=dirpe0xb9q8qiLsFr0=vr0=vr0dc8meaabaqaciaacaGaaeqabaqabeGadaaakeaadaqdbaqaaiabfs5aebaaaaa@2E24@**[ug/m^**3**^]**	**Polynomial **s¯hour MathType@MTEF@5@5@+=feaafiart1ev1aaatCvAUfKttLearuWrP9MDH5MBPbIqV92AaeXatLxBI9gBaebbnrfifHhDYfgasaacH8akY=wiFfYdH8Gipec8Eeeu0xXdbba9frFj0=OqFfea0dXdd9vqai=hGuQ8kuc9pgc9s8qqaq=dirpe0xb9q8qiLsFr0=vr0=vr0dc8meaabaqaciaacaGaaeqabaqabeGadaaakeaacuWGZbWCgaqeamaaBaaaleaacqWGObaAcqWGVbWBcqWG1bqDcqWGYbGCaeqaaaaa@33FF@**[ug/m^**3**^]**
**200**	0.141	0.143	0.140	0.142
**400**	0.309	0.346	0.307	0.345
**800**	0.473	0.682	0.465	0.678
**1600**	0.394	1.119	0.342	1.125

The interpolated surface should preferably be as close as possible to the modelled surface of the fine grid. As can be seen in Table 1 the differences and standard deviations between the two interpolation methods are quite similar. Both the mean and the standard deviation are slightly better for the polynomial interpolation method than the bilinear.

Studying the different methods visually reveals, however, that the interpolation methods differ spatially. As can be seen from the polynomial interpolation shown in Figure 4, this method gives low values less than zero. This is caused by the fact that polynomial interpolation of degree three can model local maxima/minima between the input values where the contrast for the modelled values is high (this implies that the parameter *a*_3 _in Eq. 2 is large if the input values vary much). In Figure 4, the local minima for the polynomial interpolation are seen as black dots close to areas with high concentrations (i.e. bright areas).

Although the polynomial surface seems to follow the fine surface slightly better on average than the bilinear interpolation, we chose to use the bilinear interpolation method for this study (and recommend bilinear interpolation be used in future studies). The main reason is that polynomial interpolation can produce poor-quality output if there is high contrast in the input data.

### Comparison of interpolated values and modelled values

**Figure 5:**
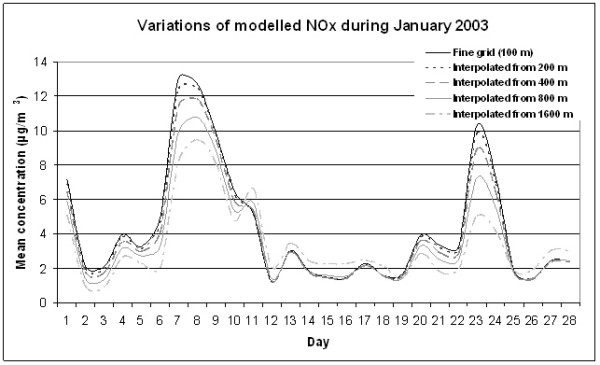
Time series of NO_x _concentrations (μg/m^3^) for different resolutions interpolated to 100 metres by bilinear interpolation. The graph shows the mean value for all cells.

To estimate the loss of information using a coarse grid instead of a fine grid we compared the fine grid and the interpolated coarse grids by plotting their daily mean NO_x _concentrations against each other. In Figure 5 the modelled values for the 100-metre grid (fine grid) and for the interpolated coarse grids (200, 400, 800 and 1600 metres) for the urban study site are plotted in a time series of daily values. As can be seen in Table 2 and Figure 5 almost all of the coarser grids, except for the 1600-m interpolated grid, gave lower or equal concentrations when they were interpolated to the same resolution as the fine grid. The grid with the coarsest resolution, 1600 metres, overestimates the concentrations during periods with low concentration. The accuracy and fit to the fine grid seem to be dependent on the resolution, the higher resolution, the better the accuracy compared to the fine grid.

**Table 2:** Mean (Δ¯¯
MathType@MTEF@5@5@+=feaafiart1ev1aaatCvAUfKttLearuWrP9MDH5MBPbIqV92AaeXatLxBI9gBaebbnrfifHhDYfgasaacH8akY=wiFfYdH8Gipec8Eeeu0xXdbba9frFj0=OqFfea0dXdd9vqai=hGuQ8kuc9pgc9s8qqaq=dirpe0xb9q8qiLsFr0=vr0=vr0dc8meaabaqaciaacaGaaeqabaqabeGadaaakeaadaqdbaqaaiabfs5aebaaaaa@2E24@) and standard deviation (s¯hour
 MathType@MTEF@5@5@+=feaafiart1ev1aaatCvAUfKttLearuWrP9MDH5MBPbIqV92AaeXatLxBI9gBaebbnrfifHhDYfgasaacH8akY=wiFfYdH8Gipec8Eeeu0xXdbba9frFj0=OqFfea0dXdd9vqai=hGuQ8kuc9pgc9s8qqaq=dirpe0xb9q8qiLsFr0=vr0=vr0dc8meaabaqaciaacaGaaeqabaqabeGadaaakeaacuWGZbWCgaqeamaaBaaaleaacqWGObaAcqWGVbWBcqWG1bqDcqWGYbGCaeqaaaaa@33FF@, s¯day
 MathType@MTEF@5@5@+=feaafiart1ev1aaatCvAUfKttLearuWrP9MDH5MBPbIqV92AaeXatLxBI9gBaebbnrfifHhDYfgasaacH8akY=wiFfYdH8Gipec8Eeeu0xXdbba9frFj0=OqFfea0dXdd9vqai=hGuQ8kuc9pgc9s8qqaq=dirpe0xb9q8qiLsFr0=vr0=vr0dc8meaabaqaciaacaGaaeqabaqabeGadaaakeaacuWGZbWCgaqeamaaBaaaleaacqWGKbazcqWGHbqycqWG5bqEaeqaaaaa@3276@, s¯week
 MathType@MTEF@5@5@+=feaafiart1ev1aaatCvAUfKttLearuWrP9MDH5MBPbIqV92AaeXatLxBI9gBaebbnrfifHhDYfgasaacH8akY=wiFfYdH8Gipec8Eeeu0xXdbba9frFj0=OqFfea0dXdd9vqai=hGuQ8kuc9pgc9s8qqaq=dirpe0xb9q8qiLsFr0=vr0=vr0dc8meaabaqaciaacaGaaeqabaqabeGadaaakeaacuWGZbWCgaqeamaaBaaaleaacqWG3bWDcqWGLbqzcqWGLbqzcqWGRbWAaeqaaaaa@33DB@) of differences in concentrations of NO_X _(μg/m^3^) between the interpolated coarse resolutions and the fine grid for the whole month of January.

**Resolution**	Δ¯¯ MathType@MTEF@5@5@+=feaafiart1ev1aaatCvAUfKttLearuWrP9MDH5MBPbIqV92AaeXatLxBI9gBaebbnrfifHhDYfgasaacH8akY=wiFfYdH8Gipec8Eeeu0xXdbba9frFj0=OqFfea0dXdd9vqai=hGuQ8kuc9pgc9s8qqaq=dirpe0xb9q8qiLsFr0=vr0=vr0dc8meaabaqaciaacaGaaeqabaqabeGadaaakeaadaqdbaqaaiabfs5aebaaaaa@2E24@	s¯hour MathType@MTEF@5@5@+=feaafiart1ev1aaatCvAUfKttLearuWrP9MDH5MBPbIqV92AaeXatLxBI9gBaebbnrfifHhDYfgasaacH8akY=wiFfYdH8Gipec8Eeeu0xXdbba9frFj0=OqFfea0dXdd9vqai=hGuQ8kuc9pgc9s8qqaq=dirpe0xb9q8qiLsFr0=vr0=vr0dc8meaabaqaciaacaGaaeqabaqabeGadaaakeaacuWGZbWCgaqeamaaBaaaleaacqWGObaAcqWGVbWBcqWG1bqDcqWGYbGCaeqaaaaa@33FF@	s¯day MathType@MTEF@5@5@+=feaafiart1ev1aaatCvAUfKttLearuWrP9MDH5MBPbIqV92AaeXatLxBI9gBaebbnrfifHhDYfgasaacH8akY=wiFfYdH8Gipec8Eeeu0xXdbba9frFj0=OqFfea0dXdd9vqai=hGuQ8kuc9pgc9s8qqaq=dirpe0xb9q8qiLsFr0=vr0=vr0dc8meaabaqaciaacaGaaeqabaqabeGadaaakeaacuWGZbWCgaqeamaaBaaaleaacqWGKbazcqWGHbqycqWG5bqEaeqaaaaa@3276@	s¯week MathType@MTEF@5@5@+=feaafiart1ev1aaatCvAUfKttLearuWrP9MDH5MBPbIqV92AaeXatLxBI9gBaebbnrfifHhDYfgasaacH8akY=wiFfYdH8Gipec8Eeeu0xXdbba9frFj0=OqFfea0dXdd9vqai=hGuQ8kuc9pgc9s8qqaq=dirpe0xb9q8qiLsFr0=vr0=vr0dc8meaabaqaciaacaGaaeqabaqabeGadaaakeaacuWGZbWCgaqeamaaBaaaleaacqWG3bWDcqWGLbqzcqWGLbqzcqWGRbWAaeqaaaaa@33DB@
**Urban study site: Lund**				
**200 m**	0.14	0.87	0.45	0.22
**400 m**	0.38	1.63	0.80	0.40
**800 m**	0.70	3.00	1.36	0.69
**1600 m**	0.96	5.52	2.52	1.33
**Rural study site: Genarp**				
**200 m**	0.03	0.16	0.08	0.04
**400 m**	0.09	0.27	0.14	0.07
**800 m**	0.17	0.46	0.23	0.10
**1600 m**	0.27	0.71	0.36	0.15

**Figure 6:**
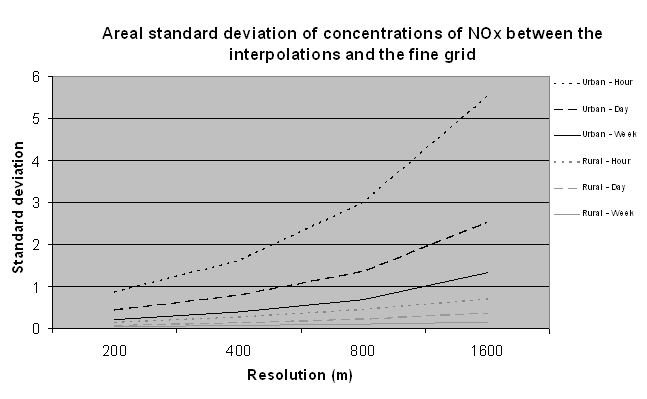
Areal standard deviation of NO_x _concentrations (μg/m^3^) for different resolutions interpolated to 100 metres by bilinear interpolation. The graphs show the values for the urban and rural study sites.

Studying the standard deviations in Table 2 and Figure 6 reveals that when concentration values are aggregated in time (i.e. hours, days and weeks) the deviation and disagreement between the different resolutions is considerably reduced. It is also quite obvious that the standard deviation is much lower for the rural study site than for the urban one.

### Urban study site – Lund

**Figure 7:**
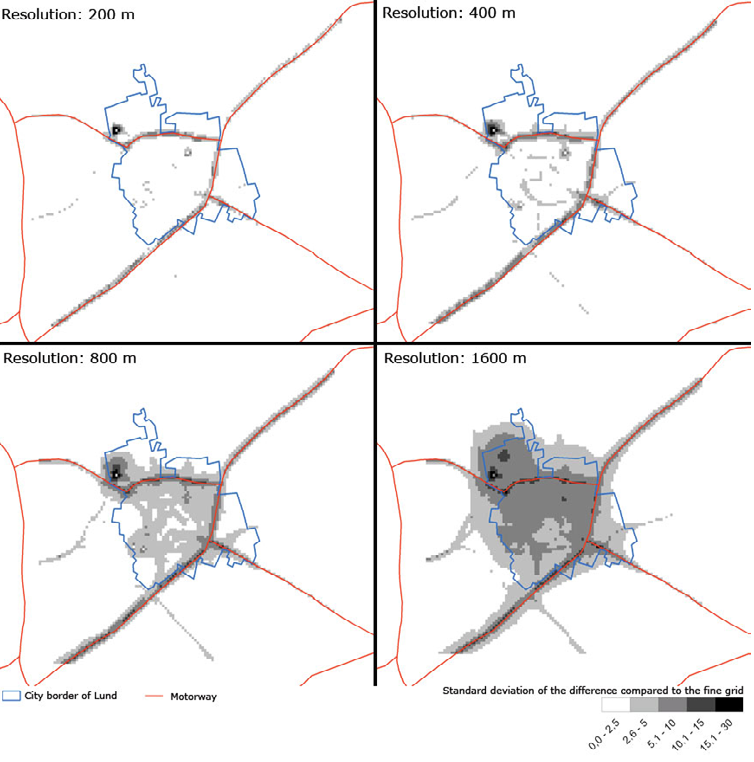
Spatial distribution of the standard deviation of the discrepancy between the coarse grids and the fine grid (100 metres) (s¯dayi,j
 MathType@MTEF@5@5@+=feaafiart1ev1aaatCvAUfKttLearuWrP9MDH5MBPbIqV92AaeXatLxBI9gBaebbnrfifHhDYfgasaacH8akY=wiFfYdH8Gipec8Eeeu0xXdbba9frFj0=OqFfea0dXdd9vqai=hGuQ8kuc9pgc9s8qqaq=dirpe0xb9q8qiLsFr0=vr0=vr0dc8meaabaqaciaacaGaaeqabaqabeGadaaakeaacuWGZbWCgaqeamaaDaaaleaacqWGKbazcqWGHbqycqWG5bqEaeaacqWGPbqAcqGGSaalcqWGQbGAaaaaaa@360F@ in Eq.n 4) of NO_x _[μg/m^3^] for the urban study site.

The spatial distribution of standard deviations of differences in daily concentrations (s¯dayi,j
 MathType@MTEF@5@5@+=feaafiart1ev1aaatCvAUfKttLearuWrP9MDH5MBPbIqV92AaeXatLxBI9gBaebbnrfifHhDYfgasaacH8akY=wiFfYdH8Gipec8Eeeu0xXdbba9frFj0=OqFfea0dXdd9vqai=hGuQ8kuc9pgc9s8qqaq=dirpe0xb9q8qiLsFr0=vr0=vr0dc8meaabaqaciaacaGaaeqabaqabeGadaaakeaacuWGZbWCgaqeamaaDaaaleaacqWGKbazcqWGHbqycqWG5bqEaeaacqWGPbqAcqGGSaalcqWGQbGAaaaaaa@360F@) between different resolutions of modelled daily concentrations for the urban study area can be seen in Figure 7. The dissimilarity at lower resolutions is quite apparent, and varies between 0 and 30 μg/m^3^. The largest divergence is seen in areas of high contrast, such as along roads, near combustion sources and in the city centre. For the resolution of 200-metres the divergence is largest near the motorways, with values up to approximately 5 μg/m^3^, and near combustion sources, 2.60–30 μg/m^3^. For the 400-metre grid the values begin to diverge near major roads (s¯dayi,j
 MathType@MTEF@5@5@+=feaafiart1ev1aaatCvAUfKttLearuWrP9MDH5MBPbIqV92AaeXatLxBI9gBaebbnrfifHhDYfgasaacH8akY=wiFfYdH8Gipec8Eeeu0xXdbba9frFj0=OqFfea0dXdd9vqai=hGuQ8kuc9pgc9s8qqaq=dirpe0xb9q8qiLsFr0=vr0=vr0dc8meaabaqaciaacaGaaeqabaqabeGadaaakeaacuWGZbWCgaqeamaaDaaaleaacqWGKbazcqWGHbqycqWG5bqEaeaacqWGPbqAcqGGSaalcqWGQbGAaaaaaa@360F@ approximately 5–10 μg/m^3^). For the lower resolutions of 800- and 1600-metres calculations fail to reflect the correct concentrations in the city centre, and for the coarsest resolution (1600 metres) the divergence for the entire city of Lund is 5.84 μg/m^3^.

### Rural study site – Genarp

**Figure 8:**
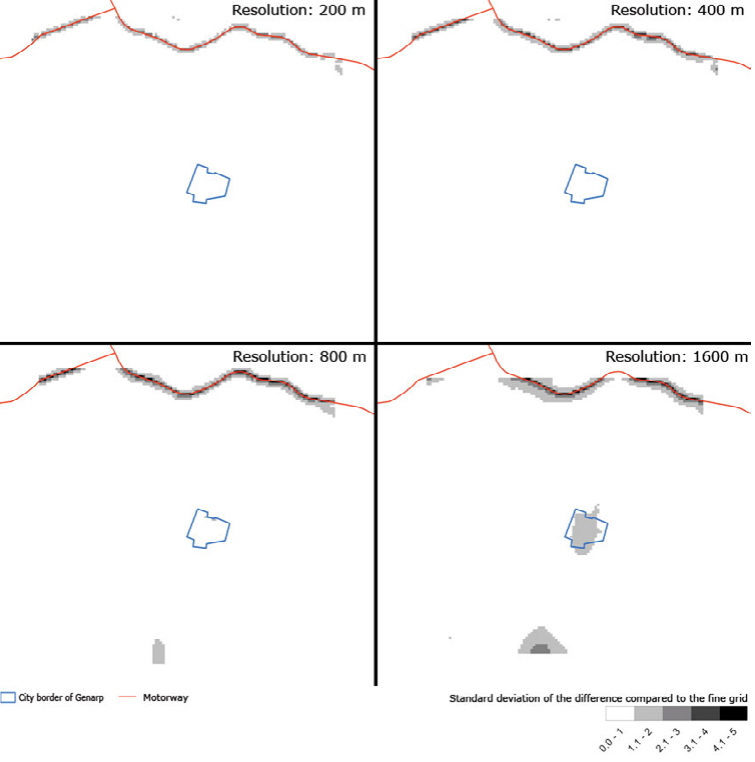
Spatial distribution of the standard deviation of the discrepancy between the coarse grids and the fine grid (100 metres) (equal to s¯dayi,j
 MathType@MTEF@5@5@+=feaafiart1ev1aaatCvAUfKttLearuWrP9MDH5MBPbIqV92AaeXatLxBI9gBaebbnrfifHhDYfgasaacH8akY=wiFfYdH8Gipec8Eeeu0xXdbba9frFj0=OqFfea0dXdd9vqai=hGuQ8kuc9pgc9s8qqaq=dirpe0xb9q8qiLsFr0=vr0=vr0dc8meaabaqaciaacaGaaeqabaqabeGadaaakeaacuWGZbWCgaqeamaaDaaaleaacqWGKbazcqWGHbqycqWG5bqEaeaacqWGPbqAcqGGSaalcqWGQbGAaaaaaa@360F@ in Eq. 4) of NO_x _[μg/m^3^] for the rural study site.

The spatial distribution of the standard deviation of differences between different resolutions of modelled daily concentrations for the rural study area can be seen in Figure 8. The dissimilarity at lower resolutions is not as apparent as for the urban study area. The divergence varies between 0 and 5 μg/m^3^, but for all the resolutions most of the area has a standard deviation of less than 1 μg/m^3^. As in the case of the urban study area, the highest contrasts occur along roads and near the village centre. For the 200-m resolution the standard deviation of the difference is largest near the motorway 0 – 3 μg/m^3 ^and the standard deviation near the village of Genarp is well above 1 μg/m^3^. For the 400- and 800-metre grids the changes in estimation along the motorway are somewhat more apparent and the standard deviation rises to a maximum of approximately 5 μg/m^3^. Using a resolution of 1600 metres the daily standard deviation for the village centre of Genarp increases to levels between 1.1 and 2 μg/m^3^.

As can be seen from Table 2 and Figure 5 there is a systematic difference between the values from the fine grid and the interpolated values from the coarse grids. The larger the cell size, the lower the NO_x_value. It should be noted that this is not due to the interpolation but the dispersion modelling. This can be illustrated by studying the original modelled values. The mean values for all modelled points (for all cells and time intervals); were computed and denoted *Mean100*, etc. The difference between the grids was then calculated to obtain: *Mean100*-*Mean200 *= 0.14 μg/m^3^, *Mean100*-*Mean400 *= 0.32 μg/m^3^, *Mean100*-*Mean800 *= 0.52 μg/m^3^, and *Mean100*-*Mean1600 *= 0.52 μg/m^3^. These values show a clear resemblance to the values in Table 2.

### Preference of resolution

It is important to take into consideration the properties of the modelled area into consideration. Concentrations modelled in urban areas with coarser resolution than 400 metres seem to generate larger error estimates in areas with high contrasts. However, for rural areas, where the variations in air pollutants are much lower, even a grid size of 1600 metres seems to generate reasonable results. However, most individuals in Scania live in urban areas where both levels and gradients of air pollutants are high. When conducting exposure studies in these areas too low a resolution might fail to reflect these variations, thus giving incorrect exposure estimates for the inhabitants. Therefore, the results from the urban study site should have a greater impact on the choice of resolution. If data are to be stored as hourly values a resolution of, at least, 400 metres is preferable as lower resolutions will yield too high deviations in the cities and in areas with high gradients. However, this may lead to a huge increase in the need for generation and storage of data. One option could therefore be to generate a grid for the entire region of Scania with a resolution of 800–1600 metres, and in addition to this generate concentration grids with higher resolution, at least 400 metres, for the larger urban areas. This could lead to calculated concentrations in areas along major roads in the rural areas having a larger error than the rest of the region. The advantages, on the other hand, would be that the data storage and computer capacity needed would decrease considerably, while exposure estimates for the vast majority of the population in the region would be accurate.

## Conclusion

When establishing a pollutant database certain criteria must be taken into consideration. The fact that a pollutant database has a fixed resolution in time and space makes the choice of resolution for these two parameters critical for the future use of the database. The aim of this study was to investigate the optimal spatial resolution with respect to temporal resolution for future epidemiological studies in Scania, Sweden. The pollutant database of NO_x _values will be used mainly for epidemiological studies with durations of days, weeks or even longer periods. Some studies might model exposure for a dynamic population (e.g. modelling being at work and at home), and therefore we need an hourly time resolution in the pollutant database. However, since the duration of the studies is at least one day the spatial resolution should be set in relation to this time resolution.

The standard deviation for the accuracy of the modelled daily mean values of air pollutants is approximately 6 μg/m^3^; a value that could be decreased by about 50% if the extreme values are removed. Previous studies [5] have given similar accuracy estimations. The choice of spatial resolution should not considerably deteriorate the accuracy of the modelled values; therefore, we believe that an error due to coarse resolution greater than 1 μg/m^3 ^is inadvisable. Based on Table 2 we conclude that for urban areas a spatial resolution of 200–400 m is suitable; and for rural areas the spatial resolution could be coarser (about 1600 m). This implies that we should develop a pollutant database that allows different spatial resolution for urban and rural areas.

## Methods

The procedure used in this study is described below and follows the study design illustrated in Figure 2.

### Comparison of modelled values and observed values

NO_x _values were modelled using a dispersion software and an emission source database for air pollutants. This database was constructed by the GIS Centre, Lund University, supported by the Swedish National Air Pollution and Health Effects Programme (SNAP)[5]. The database includes emission data for all major sources for the years 2000–2004; the main pollutants included are nitrogen oxides (NO_x_) and particular matter. The emission source database covers the whole Öresund region, where Scania, Zealand (Denmark) and the sea around Scania. The purpose of extending the area is to obtain more precise description of air quality in Scania, since emissions from Zealand and shipping affect the air quality in the western part of Scania. The most important sources in the database are road traffic, shipping, aviation, rail transport, industry, power plants, small-scale heating and machinery. Information, statistics and emissions were collected from official sources. The database includes data on about 23,000 road sources, 500 point sources and nearly 100 area sources.

For modelling we used the dispersion software Enviman [17]. Enviman estimates the pollutant values over a grid in which all emission sources are located in the centre of the cell. A box model is used to compute the contribution to the cell in which the emission sources are located. The contribution to other cells is calculated using a Gaussian dispersion method [18]. Enviman has a default time resolution of one hour. To perform dispersion calculations a meteorological dataset is needed. In this study we used observed meteorological data, such as wind speed, wind direction, temperature and solar radiation. The meteorological observations were made on a 24 m high mast, in an open field a few kilometres from central Malmö. In the calculations, we allowed the plume from each source inside the calculation area travel 6 hours. After six hours, we assumed that the concentration was so low that it had no significant effect on the air pollution levels.

Enviman dispersion software and the emission source database were used to estimate the hourly NO_x _values at the location of the meteorological station in Malmö during four weeks in January 2003. Hourly NO_x _values for the same time period were also collected from the meteorological station. The measurements were performed by the local environment protection agency in Malmö and the measuring technique was chemiluminescence. Temporal variations, means and standard deviation between the modelled and measured concentrations were then evaluated.

### Generation of pollutant fields with a time resolution of one hour

Enviman was used to produce five pollutant fields of NO_x _for each of the two study sites, with spatial resolutions of 100 m (denoted *fine grid*), 200 m, 400 m, 800 m and 1.6 km (denoted *coarse grids*). The time resolution for all pollutant fields was one hour and the total time modelled was four weeks in January 2003. In the modelling we used meteorological data from Malmö. It should be noted that the modelling of these pollutant fields does not include background emissions; the reason being that the aim was only to estimate the effect of using different spatial resolutions and for this estimation the background emissions has no effect.

**Figure 9:**
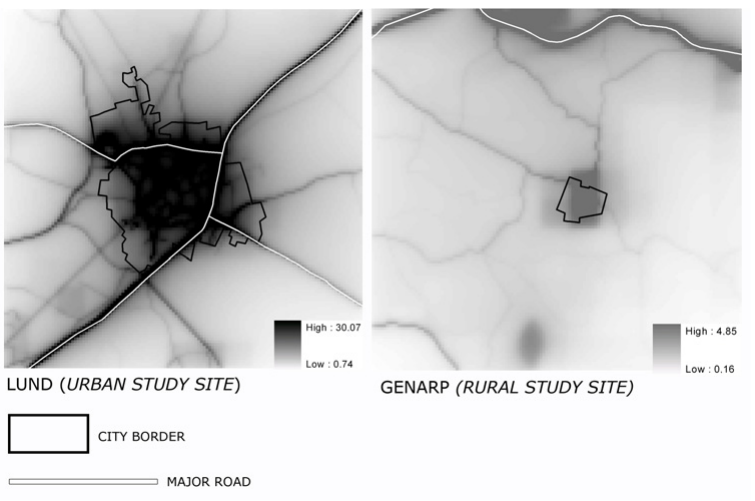
Illustration of the modelled NO_x _values (μg/m^3^) at the urban study site (Lund) and the rural study site (Genarp).

Figure 9 illustrates the 100 m pollutant fields computed for the two study sites. For the urban study site the mean level of NO_x _(from local sources during the study period January 2003) varies between 0.74 and 30.07 μg/m^3^. It can be seen that the highest values are along the motorways and other major roads, and in the city centre. For the rural study site the level, as well as the gradient, of air pollution is low in comparison with the urban study site; the mean concentrations of NO_x _in the study area during January 2003 were between 0.16 and 4.85 μg/m^3^.

### Generation of pollutant fields with time resolutions of one day and one week

From the one-hour data we computed pollutant fields of NO_X _values with temporal resolutions of *one day *and *one week*. This step was simply performed by taking the mean values of the one-hour data.

### Interpolation of the coarse pollutant fields

**Figure 10:**
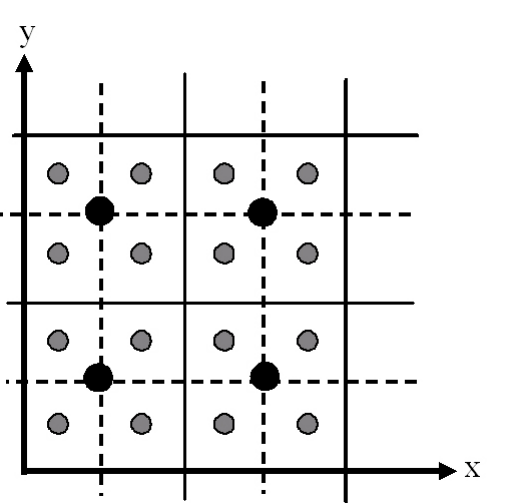
The black dots indicate the centre points in the coarse grid (the solid line); the grey smaller dots show the centre points of the fine (or interpolated) grid (dashed line).

To estimate the loss of information when using a coarser grid it is necessary to know the NO_X _values for the same locations for all pollutant fields. The basic approach used was to estimate the NO_X _values in the coarse grid for all the points in the fine grid; this was performed by interpolation. The dispersion software (Enviman) estimates the NO_X _value at the centre of each cell in the grid. From Figure 10, we can see that there are no common points modelled in both the fine grid and in the coarse grids (if the cell sizes differ by a factor of 2 and the grids have the same extent).

The ideal interpolation method for our application should generate a continuous surface that passes through all the modelled values (i.e., the input values for the interpolation), this and the interpolated surface should be as close as possible to the modelled surface. In this study, we tested two interpolation methods: *bilinear interpolation *and *polynomial interpolation *[19-21].

**Figure 11:**
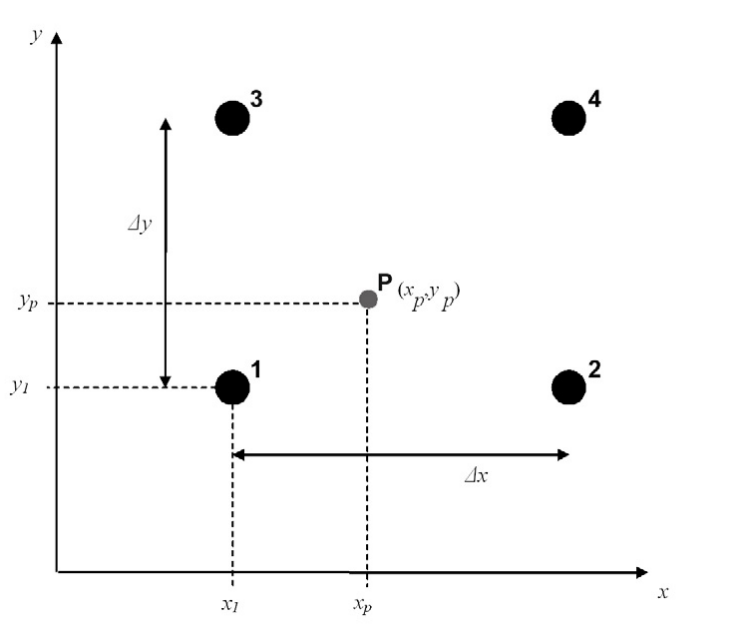
Bilinear interpolation. The value at point P (with coordinates x_p_, y_p_) is interpolated from the values at points 1–4.

Bilinear interpolation requires that the input points be given in a regular grid, and is defined in Eq. 1 (using the notation in Figure 11):

*z *(*x*_p_, *y*_p_) = *z*_1 _+ (*z*_2 _- *z*_1_)·*w *+ (*z*_3 _- *z*_1_)·*u *+ (*z*_1 _- *z*_2 _-*z*_3 _+ *z*_4_)·*uw*

where

*z*(*x*_*p*_, *y*_*p*_) = interpolated value at the point *x*_*p*_, *y*_*p *_(i.e. the point at which the value is to be interpolated),

z_1_, z_2_, z_3_, z_4 _= values of the four closest points (to *x*_*p*_, *y*_*p*_) in the coarse grid,

*w *= (*x*_*p *_- *x*_*1*_)/Δ*x*,

*u *= (*y*_*p *_- *y*_*1*_)/Δ*y *and

Δ*x*, Δ*y *= the spatial resolution of the coarse grid.

Bilinear interpolation has the advantage that it is simple and creates a continuous surface that passes through all the input points (points 1–4 in Eq.1). But it is not capable of modelling extreme values between the input points.

**Figure 12:**
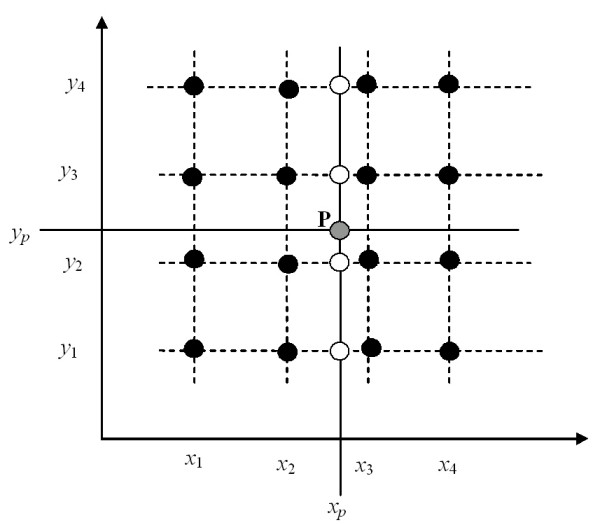
Interpolation of the value at point P (x_p_, y_p_) (greydot) by a succession of polynomial interpolations. First, the values at points (y_i_, x_p_) (i = 1,2,3,4) (white dots) are interpolated (in the x-direction) using Eq. 2. These new values are then used as input to interpolate in the y-direction to determine the value at the point P (x_p_, y_p_).

Polynomial interpolation can be implemented by computing a two-dimensional polynomial surface. However, this is too computationally intensive for our application (we have to interpolate around 100 million points) and therefore we decided to use a succession of one-dimensional interpolations. We used polynomials of degree three and 4 × 4 input points in a regular grid (equal to the 16 closest points in the coarse grid) for the interpolation. The approach adopted was to start computing a one-dimensional polynomial for a fixed *y*-value (*y*_1_) using Eq. 2 (with the notation in Figure 12):

*z*(*x*_*p*_|*y *= *y*_1_) = *a*_0 _+ *a*_1_·*x*_*p *_+ *a*_2_·*x*_*p*_^2 ^+ *a*_3_·*x*_*p*_^3 ^

Since only four values (along the line *y *= *y*_1_) are used to estimate the polynomial parameters (*a*_*i*_) there is a unique solution (in reality the polynomial is written somewhat differently, (see [21]), but here we prefer this simple form for clarity). Equation 2 is then applied for the other three *y*-values; this implies that we have four estimations of *z *for *x *= *x*_*p*_. Finally, these values are used to interpolate the point (*x*_*p*_, *y*_*p*_) using a polynomial in the *y*-direction (Figure 12).

Polynomial interpolation provides a continuous surface as long as the same input points are used. However along the boundary lines where the input points are changed the surface is discontinuous. Polynomial interpolation (of degree three) is capable of modelling extreme values between the input values. This can be both an advantage and a disadvantage. For some types of surface characteristics the extreme values will decrease the difference between a modelled surface and an interpolated surface; for other types the difference will increase.

### Comparison of the fine grid and the coarse grids

Interpolation provides pollutant fields with values for the same points. For the fine grid the values are modelled and for the coarse grid the values are interpolated. Furthermore, we have pollutant fields with time resolutions of *one hour*, *one day *and *one week*.

A number of methods are available to compare the values from the grids. Of special interest for our objective is the discrepancy between the values from the modelled fine grid and the interpolated values from the coarse grids. The discrepancy is estimated for each cell by its mean value and the standard deviation. The mean value of the discrepancy for each cell (Δ¯i,j
 MathType@MTEF@5@5@+=feaafiart1ev1aaatCvAUfKttLearuWrP9MDH5MBPbIqV92AaeXatLxBI9gBaebbnrfifHhDYfgasaacH8akY=wiFfYdH8Gipec8Eeeu0xXdbba9frFj0=OqFfea0dXdd9vqai=hGuQ8kuc9pgc9s8qqaq=dirpe0xb9q8qiLsFr0=vr0=vr0dc8meaabaqaciaacaGaaeqabaqabeGadaaakeaacuqHuoargaqeamaaCaaaleqabaGaemyAaKMaeiilaWIaemOAaOgaaaaa@31EF@) is estimated by:

Δ¯i,j=1Thour⋅∑t=1ThourFineNOXhour,ti,j−CoarseNOXhour,ti,j
 MathType@MTEF@5@5@+=feaafiart1ev1aaatCvAUfKttLearuWrP9MDH5MBPbIqV92AaeXatLxBI9gBaebbnrfifHhDYfgasaacH8akY=wiFfYdH8Gipec8Eeeu0xXdbba9frFj0=OqFfea0dXdd9vqai=hGuQ8kuc9pgc9s8qqaq=dirpe0xb9q8qiLsFr0=vr0=vr0dc8meaabaqaciaacaGaaeqabaqabeGadaaakeaacuqHuoargaqeamaaCaaaleqabaGaemyAaKMaeiilaWIaemOAaOgaaOGaeyypa0ZaaSaaaeaacqaIXaqmaeaacqWGubavdaWgaaWcbaGaemiAaGMaem4Ba8MaemyDauNaemOCaihabeaaaaGccqGHflY1daaeWbqaaiabdAeagjabdMgaPjabd6gaUjabdwgaLjabd6eaojabd+eapjabdIfaynaaDaaaleaacqWGObaAcqWGVbWBcqWG1bqDcqWGYbGCcqGGSaalcqWG0baDaeaacqWGPbqAcqGGSaalcqWGQbGAaaGccqGHsislcqWGdbWqcqWGVbWBcqWGHbqycqWGYbGCcqWGZbWCcqWGLbqzcqWGobGtcqWGpbWtcqWGybawdaqhaaWcbaGaemiAaGMaem4Ba8MaemyDauNaemOCaiNaeiilaWIaemiDaqhabaGaemyAaKMaeiilaWIaemOAaOgaaaqaaiabdsha0jabg2da9iabigdaXaqaaiabdsfaunaaBaaameaacqWGObaAcqWGVbWBcqWG1bqDcqWGYbGCaeqaaaqdcqGHris5aaaa@7671@

where:

*i*, *j *denotes that the cell in row *i *and column *j *in the grid,

*T*_*hour *_is the number of time intervals for a spatial resolution of one hour,

*t *denotes the time interval,

FineNOXhour,ti,j
 MathType@MTEF@5@5@+=feaafiart1ev1aaatCvAUfKttLearuWrP9MDH5MBPbIqV92AaeXatLxBI9gBaebbnrfifHhDYfgasaacH8akY=wiFfYdH8Gipec8Eeeu0xXdbba9frFj0=OqFfea0dXdd9vqai=hGuQ8kuc9pgc9s8qqaq=dirpe0xb9q8qiLsFr0=vr0=vr0dc8meaabaqaciaacaGaaeqabaqabeGadaaakeaacqWGgbGrcqWGPbqAcqWGUbGBcqWGLbqzcqWGobGtcqWGpbWtcqWGybawdaqhaaWcbaGaemiAaGMaem4Ba8MaemyDauNaemOCaiNaeiilaWIaemiDaqhabaGaemyAaKMaeiilaWIaemOAaOgaaaaa@410F@ are the NO_X _values for time interval *t *and cell *i*, *j *in the fine grid, and

CoarseNOXhour,ti,j
 MathType@MTEF@5@5@+=feaafiart1ev1aaatCvAUfKttLearuWrP9MDH5MBPbIqV92AaeXatLxBI9gBaebbnrfifHhDYfgasaacH8akY=wiFfYdH8Gipec8Eeeu0xXdbba9frFj0=OqFfea0dXdd9vqai=hGuQ8kuc9pgc9s8qqaq=dirpe0xb9q8qiLsFr0=vr0=vr0dc8meaabaqaciaacaGaaeqabaqabeGadaaakeaacqWGdbWqcqWGVbWBcqWGHbqycqWGYbGCcqWGZbWCcqWGLbqzcqWGobGtcqWGpbWtcqWGybawdaqhaaWcbaGaemiAaGMaem4Ba8MaemyDauNaemOCaiNaeiilaWIaemiDaqhabaGaemyAaKMaeiilaWIaemOAaOgaaaaa@43D7@ are the NO_X _values for time interval *t *and cell *i*,*j *in the interpolated coarse grid.

The mean value will be the same regardless of whether we study hourly, daily or weekly NO_X _values (since the daily and weekly data were computed as mean values from the hourly data). Behind this lies an approximation that the mean values of the discrepancies are constant throughout the day and between different days. This approximation is fairly good.

The standard deviation will vary depending on whether we study hourly, daily or weekly data. For each cell and time resolution, the standard deviation (sxi,j
 MathType@MTEF@5@5@+=feaafiart1ev1aaatCvAUfKttLearuWrP9MDH5MBPbIqV92AaeXatLxBI9gBaebbnrfifHhDYfgasaacH8akY=wiFfYdH8Gipec8Eeeu0xXdbba9frFj0=OqFfea0dXdd9vqai=hGuQ8kuc9pgc9s8qqaq=dirpe0xb9q8qiLsFr0=vr0=vr0dc8meaabaqaciaacaGaaeqabaqabeGadaaakeaacqWGZbWCdaqhaaWcbaGaemiEaGhabaGaemyAaKMaeiilaWIaemOAaOgaaaaa@3359@) is given by:

sxi,j=1Tx−1⋅∑t=1Tx(Δx,ti,j−Δ¯i,j)2
 MathType@MTEF@5@5@+=feaafiart1ev1aaatCvAUfKttLearuWrP9MDH5MBPbIqV92AaeXatLxBI9gBaebbnrfifHhDYfgasaacH8akY=wiFfYdH8Gipec8Eeeu0xXdbba9frFj0=OqFfea0dXdd9vqai=hGuQ8kuc9pgc9s8qqaq=dirpe0xb9q8qiLsFr0=vr0=vr0dc8meaabaqaciaacaGaaeqabaqabeGadaaakeaacqWGZbWCdaqhaaWcbaGaemiEaGhabaGaemyAaKMaeiilaWIaemOAaOgaaOGaeyypa0ZaaOaaaeaadaWcaaqaaiabigdaXaqaaiabdsfaunaaBaaaleaacqWG4baEaeqaaOGaeyOeI0IaeGymaedaaiabgwSixpaaqahabaGaeiikaGIaeuiLdq0aa0baaSqaaiabdIha4jabcYcaSiabdsha0bqaaiabdMgaPjabcYcaSiabdQgaQbaakiabgkHiTiqbfs5aezaaraWaaWbaaSqabeaacqWGPbqAcqGGSaalcqWGQbGAaaGccqGGPaqkdaahaaWcbeqaaiabikdaYaaaaeaacqWG0baDcqGH9aqpcqaIXaqmaeaacqWGubavcqWG4baEa0GaeyyeIuoaaSqabaaaaa@56DD@

where:

*x *denotes the time resolution (*hour*, *day *or *week*), and

Δx,ti,j=FineNOXx,ti,j−CoarseNOXx,ti,j.
 MathType@MTEF@5@5@+=feaafiart1ev1aaatCvAUfKttLearuWrP9MDH5MBPbIqV92AaeXatLxBI9gBaebbnrfifHhDYfgasaacH8akY=wiFfYdH8Gipec8Eeeu0xXdbba9frFj0=OqFfea0dXdd9vqai=hGuQ8kuc9pgc9s8qqaq=dirpe0xb9q8qiLsFr0=vr0=vr0dc8meaabaqaciaacaGaaeqabaqabeGadaaakeaacqqHuoardaqhaaWcbaGaemiEaGNaeiilaWIaemiDaqhabaGaemyAaKMaeiilaWIaemOAaOgaaOGaeyypa0JaemOrayKaemyAaKMaemOBa4MaemyzauMaemOta4Kaem4ta8KaemiwaG1aa0baaSqaaiabdIha4jabcYcaSiabdsha0bqaaiabdMgaPjabcYcaSiabdQgaQbaakiabgkHiTiabdoeadjabd+gaVjabdggaHjabdkhaYjabdohaZjabdwgaLjabd6eaojabd+eapjabdIfaynaaDaaaleaacqWG4baEcqGGSaalcqWG0baDaeaacqWGPbqAcqGGSaalcqWGQbGAaaGccqGGUaGlaaa@5BD6@

Finally, we also use the mean value for all cells, i.e.:

Δ¯¯=1NM⋅∑iN∑jMΔ¯i,j
 MathType@MTEF@5@5@+=feaafiart1ev1aaatCvAUfKttLearuWrP9MDH5MBPbIqV92AaeXatLxBI9gBaebbnrfifHhDYfgasaacH8akY=wiFfYdH8Gipec8Eeeu0xXdbba9frFj0=OqFfea0dXdd9vqai=hGuQ8kuc9pgc9s8qqaq=dirpe0xb9q8qiLsFr0=vr0=vr0dc8meaabaqaciaacaGaaeqabaqabeGadaaakeaacuqHuoargaqegaqeaiabg2da9maalaaabaGaeGymaedabaGaemOta4Kaemyta0eaaiabgwSixpaaqahabaWaaabCaeaacuqHuoargaqeamaaCaaaleqabaGaemyAaKMaeiilaWIaemOAaOgaaaqaaiabdQgaQbqaaiabd2eanbqdcqGHris5aaWcbaGaemyAaKgabaGaemOta4eaniabggHiLdaaaa@4395@

s¯x=1NM∑iN∑jMsxi,j
 MathType@MTEF@5@5@+=feaafiart1ev1aaatCvAUfKttLearuWrP9MDH5MBPbIqV92AaeXatLxBI9gBaebbnrfifHhDYfgasaacH8akY=wiFfYdH8Gipec8Eeeu0xXdbba9frFj0=OqFfea0dXdd9vqai=hGuQ8kuc9pgc9s8qqaq=dirpe0xb9q8qiLsFr0=vr0=vr0dc8meaabaqaciaacaGaaeqabaqabeGadaaakeaacuWGZbWCgaqeamaaBaaaleaacqWG4baEaeqaaOGaeyypa0ZaaSaaaeaacqaIXaqmaeaacqWGobGtcqWGnbqtaaWaaabCaeaadaaeWbqaaiabdohaZnaaDaaaleaacqWG4baEaeaacqWGPbqAcqGGSaalcqWGQbGAaaaabaGaemOAaOgabaGaemyta0eaniabggHiLdaaleaacqWGPbqAaeaacqWGobGta0GaeyyeIuoaaaa@4456@

where *N*, *M *are the number of rows/columns.

### Software environment

A program in C++ was developed to compute the daily/weekly values, perform the interpolation and compute the statistics. Input data for the program were binary files containing modelled NO_X _values from the dispersion software Enviman. For the implementation of the polynomial interpolation the program utilizes functions from *Numerical Recipes in C *[21]. The time series were generated by a standard spreadsheet program (MS Excel) and the maps by a standard GIS program (ArcGIS from ESRI).

## Authors' contributions

ES: performed the analyses and parts of the programming, participated in the design of the study, and drafted the manuscript. LH: performed the main part of the programming, wrote the interpolation section and participated in the design of the study.

SG: performed the dispersion modelling.

All authors have read and approved the final manuscript.
